# Editorial: Scales validation in the context of inclusive education

**DOI:** 10.3389/fpsyg.2026.1781095

**Published:** 2026-02-16

**Authors:** Ghaleb Alnahdi, Elsayed Elshabrawi Ahmed Hassanein, Marcela Gerardina Pozas, Verena Letzel-Alt

**Affiliations:** 1 Prince Sattam Bin Abdulaziz University, Al Kharj, Saudi Arabia; 2 Qatar University, Doha, Qatar; 3 Universite du Luxembourg, Esch-sur-Alzette, Luxembourg; 4 University of Education Freiburg, Freiburg, Germany

**Keywords:** higher education, inclusive education, primary education, scales validation, secondary education, teachers

Today's society is highly diverse and this reality is mirrored also in the educational sector. Within a diverse student population the need to implement inclusive education is widely seen as “global priority” and “shared responsibility” (Ydo, 2020, p. 97). Consequently, inclusive education can be defined as the right of every person to quality education and to realize their full potential—in early childhood education, in primary and secondary education as well as in higher education (UNICEF, 2025). Inclusive education is a concept to address all learners, regardless of their learning needs. In this vein, inclusive education does not only focus on vulnerable groups, but on each and every learner (Lindmeier and Lütje-Klose, 2015).

Therefore, inclusive education is widely seen to be the most effective way to give all children and adults the chance to learn and to develop the skills they need to thrive (UNICEF, 2025). Even though inclusive education is a global goal, its implementation differs greatly between countries worldwide because every country holds different educational policies, different teacher training curricula, different educational systems and different resources that are available (Letzel-Alt and Pozas, 2023; Maulana et al., 2023). Hence, research into the field of inclusive education is inevitable to be able to document and compare different understandings and ways of implementing inclusive education in different ways.

In order to reach the common goal to adequately implement inclusive education, an evidence-based approach, which requires in-depth research, is essential. The development of international scales to measure relevant factors linked to inclusive education is inevitable for ensuring comparability, validity, and accountability across diverse educational systems. Internationally validated scales allow researchers and policymakers to systematically assess the implementation and effectiveness of inclusive education policies, identify gaps between policy and practice, and monitor progress toward global commitments such as the Sustainable Development Goals. Against this background, this Research Topic aims to provide a first step into this direction and offer a toolbox of various relevant and validated scales for investigating inclusive education.

The studies within this Research Topic have been conducted in various countries such as Saudi Arabia, Pakistan, Egypt, Jordan, Austria, Canada, Poland, Switzerland, Germany, China and Ethiopia. Thus, data from four different continents (North America, Europe, Asia, Africa) has been explored within this Research Topic. Furthermore, the Research Topic includes scales that are constructed to measure perspectives of students, teachers and university students as well as post-graduate students.

The scales within this Research Topic can be used to identify gifted students through math tests. Moreover, scales are introduced to (digitally) asses student's social-emotional skills, to assess student's classroom engagement, students' academic resilience, non-verbal communication, teachers' collective efficacy with regards to inclusive practices, teachers' attitudes toward differentiated instruction, teachers' co-constructive collaboration, teachers' ideological and political teaching ability, teachers' attitudes to inclusion and self-efficacy for inclusive practices, university students' mindsets toward language learning, undergraduate student's work engagement, and post-graduate students' admission to University.

Although the scales presented are incorporating various perspectives and examining different aspects of inclusive education, it is evident that some stakeholders' perspectives have not been addressed within the submitted scales (such as parents, principles or policy makers). Capturing multiple perspectives of relevant stakeholders should be a future aim for researchers in the field of inclusive education. Moreover, some of the scales are internationally validated and, therefore, suitable for international use (such as the TAT-DIS scale to measure teachers' attitudes toward differentiated instruction or the scale to measure teachers' collective efficacy with regards to inclusive practices). Other scales, in their current versions, are very context-specific and would require adaptation for international application (such as the PGAT to measure post-graduate students' admission to Saudi Arabian Universities).

To achieve the global goal of creating inclusive education for all within all areas of education, evidence-based research and international comparative studies are inevitable to learn from each other and collaborate, to identify and implement good practices, and to collectively compensate for insufficient resources that hinder the implementation of inclusive education in some countries through international cooperation.
